# Understanding the ecological drivers of avian influenza virus infection in wildfowl: a continental-scale study across Africa

**DOI:** 10.1098/rspb.2011.1417

**Published:** 2011-09-14

**Authors:** N. Gaidet, A. Caron, J. Cappelle, G. S. Cumming, G. Balança, S. Hammoumi, G. Cattoli, C. Abolnik, R. Servan de Almeida, P. Gil, S. R. Fereidouni, V. Grosbois, A. Tran, J. Mundava, B. Fofana, A. B. Ould El Mamy, M. Ndlovu, J. Y. Mondain-Monval, P. Triplet, W. Hagemeijer, W. B. Karesh, S. H. Newman, T. Dodman

**Affiliations:** 1 CIRAD-ES, UR AGIRS, 34398 Montpellier, France; 2 Percy FitzPatrick Institute, University of Cape Town, Rondebosch, Cape Town 7701, South Africa; 3 CIRAD-BIOS, UMR CIRAD/INRA CMAEE, 34398 Montpellier, France; 4 OIE/FAO Reference Laboratory for avian influenza and Newcastle disease, Istituto Zooprofilattico Sper.le delle Venezie, Legnaro, Italy; 5 ARC-Onderstepoort Veterinary Institute, Private Bag X5, Onderstepoort 0110, South Africa; 6 Friedrich-Loeffler-Institut, Insel Riems, Germany; 7 National University of Science and Technology, Box 939 Ascot, Bulawayo, Zimbabwe; 8 Direction Nationale des Eaux et Forêts du Mali, BP 275 Bamako, Mali; 9 Centre National d'Elevage et de Recherches Vétérinaires, BP 167 Nouakchott, Islamic Republic of Mauritania; 10 Office National de la Chasse et de la Faune Sauvage, CNERA Avifaune Migratrice, Le Sambuc, 13200 Arles, France; 11 SMBS, 1 Place de l'Amiral Courbet, 80100 Abbeville, France; 12 Oiseaux Migrateurs du Paléarctique Occidental, 5 avenue des Chasseurs, 75017 Paris, France; 13 Wetlands International, PO Box 471, 6700 AL Wageningen, The Netherlands; 14 EcoHealth Alliance, 460 West 34th Street, New York, NY 10001, USA; 15 Food and Agriculture Organisation of the United Nations, FAO, Infectious Disease Group, Rome, Italy

**Keywords:** influenza A virus, pathogen transmission, disease ecology, wild birds, tropical, migration

## Abstract

Despite considerable effort for surveillance of wild birds for avian influenza viruses (AIVs), empirical investigations of ecological drivers of AIV prevalence in wild birds are still scarce. Here we used a continental-scale dataset, collected in tropical wetlands of 15 African countries, to test the relative roles of a range of ecological factors on patterns of AIV prevalence in wildfowl. Seasonal and geographical variations in prevalence were positively related to the local density of the wildfowl community and to the wintering period of Eurasian migratory birds in Africa. The predominant influence of wildfowl density with no influence of climatic conditions suggests, in contrast to temperate regions, a predominant role for inter-individual transmission rather than transmission via long-lived virus persisting in the environment. Higher prevalences were found in *Anas* species than in non-*Anas* species even when we account for differences in their foraging behaviour (primarily dabbling or not) or their geographical origin (Eurasian or Afro-tropical), suggesting the existence of intrinsic differences between wildfowl taxonomic groups in receptivity to infection. Birds were found infected as often in oropharyngeal as in cloacal samples, but rarely for both types of sample concurrently, indicating that both respiratory and digestive tracts may be important for AIV replication.

## 1. Introduction

Understanding the influence of host ecology on the dynamics of pathogen transmission is currently recognized as fundamental to preventing and controlling wildlife infectious diseases [1,2]. Avian influenza viruses (AIVs) in wild birds have received increasing attention in recent years in response to the emergence and spread of the H5N1 highly pathogenic avian influenza (HPAI) virus across Eurasia and Africa [3,4]. Empirical investigation of the interface between the ecology and epidemiology of AIV in wild birds are, however, still in a relatively early phase of scientific exploration, and studies exploring the ecological interactions between AIV and their natural hosts are scarce [5–8]. In table 1, we present the potential ecological drivers of AIV prevalence in wild birds based on a review of our current knowledge of the mechanisms, whereby host ecology and the environment may influence AIV transmission in wild birds [1–29]. Despite the investment of considerable effort in AIV detection in wild birds, there is a lack of data-heavy empirical tests, in particular across vast geographic areas, of the influences of these ecological drivers on AIV transmission [4].

**Table 1.** Potential ecological drivers of AIV prevalence in wild birds derived from experimental and empirical findings of the ecological interactions between AIV and wildfowl.

mechanisms	epidemiological predictions^1^ and experimental^2^ or empirical^3^ findings	ecological factors of potential influence on AIV transmission
environmental transmission	^1^bird could be infected from long-lived virus persisting in the environment by drinking or feeding; infection rate depends on the virus concentration and persistence in the environment, and bird consumption rate [9]	local climate may influence the environmental persistence of AIV
	^2^AIV can remain infectious for several months in water under experimental conditions. Warmer temperatures, radiation and desiccation reduce the duration of AIV infectivity [10,11]	
	^3^mathematical models of environmental transmission capture some patterns of AIV infection dynamics in wildfowl [9,12]	
	^2^ducks can be successfully infected by contact with contaminated water [13]	species foraging behaviour may influence exposure to environmental infection
	^3^higher prevalence is commonly reported in *Anas* species of dabbling ducks compared with diving or grazing wildfowl [3,5]	
	^3^morphological trait associated with filtration of food particles (density of lamellae) has been positively associated with variations in AIV prevalence and diversity of subtypes shed in dabbling ducks [14]	

inter-individual transmission	^1^both transmission via airborne droplets or short-lived viruses shed in the environment are considered as essentially direct because they occur on the same time scale and rely on the proximity of hosts [9,12]	host density and seasonal patterns of social aggregation may influence contact rate and transmission
	^3^seasonal peak in prevalence in a wildfowl species that forage mainly on land [8], and higher AIV detection rate in respiratory than intestinal tract, support the existence of a direct airborne transmission via the respiratory route [4,8,15]	
	^1^inter-individual transmission is expected to be density-dependent since the contact rate scales with host density [16]	
	^3^northern autumn peak in AIV prevalence in ducks coincides with a seasonal social aggregation during pre-migration and migration that likely promotes contact rate and viral transmission [5,10,17,18]	

host receptivity	^1^difference in prevalence between species may result from a difference in intrinsic receptivity to AIV infection [19]	taxonomic group may influence receptivity to infection
	^3^the spectrum of AIV receptors on host cell surface vary substantially among different bird species [20]	

host susceptibility	^2^natural and experimental AIV infection stimulates the production of long-lasting AIV specific antibodies in ducks; subsequent exposure to AIV produce a boost in AIV antibody titers [21,22]	geographical range associated with migratory behaviour and age may influence previous AIV exposure hence susceptibility to re-infection
	^2^prior exposure to homo- or heterosubtypic AIV reduces the duration and concentration of viral shedding in consecutive infections, demonstrating the existence of a partial cross-protective immunity against re-infection [21,23]	
	^3^natural consecutive infections with different AIV subtypes have been reported in ducks providing evidence that a prior exposure does not fully protect against a subsequent AI virus infection with a heterosubtypic AIV [6,24]	
	^3^higher prevalence in hatch-year birds compared with after-hatch-year birds is consistently reported [5,8]	
	^3^the duration of virus shedding decreases during the northern autumn in wild ducks [6]	

population immunity	^1^the transmission rate depend on the proportion of susceptible individuals in the host population and the rate at which they experience their first infection [1,2,16]	demographic rates and seasonal peaks in prevalence may influence the turnover of susceptible hosts
	^3^prevalence strongly decline during northern autumn and winter as the proportion of immunologically naive hatch-year birds progressively decreases through infection or as result of a greater mortality rate compared with adults [5,17,18,25]	

host dispersal	^1^infected hosts shedding virus may disperse AIV as they move [2,10]	timing and range of migration may influence period and origin of virus introduction
	^2,3^experimentally [21,26] and naturally infected wildfowl [6] generally excrete AIV for one to three weeks without clinical signs or lesions	
	^3^migratory wildfowl are able to perform long-distance movements within the time frame of AIV infection [27]	
	^3^phylogenetic analysis confirms the occurrence of inter-continental exchange of AIV [28]	
	^3^phylo-geographical clusters of AIV in wildfowl across North America suggest a dominance of introduction over persistence in the interannual perpetuation of AIV [29]	

**Figure 1.**
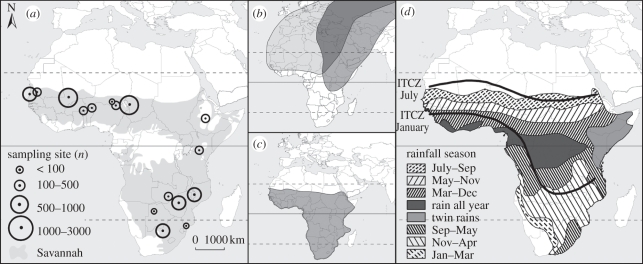
(*a*) Location of study sites (number of birds sampled); (*b*) main migratory flyways, and distribution range of Eurasian wildfowl and (*c*) Afro-tropical wildfowl in sub-Saharan Africa, adapted from [30]; (*d*) timing of the wet season and seasonal position of the Inter-Tropical Convergence Zone (ITCZ) adapted from [31].

To test a range of current assumptions and hypotheses about the ecological drivers of AIV prevalence, we investigated the relative roles of a range of ecological factors, including species traits, migration patterns, climate and seasonal fluctuations in the abundance and composition of the host community, on patterns of AIV prevalence in their main natural reservoir and wildfowl (*Anseriformes*). We explored relationships across a variety of environmental conditions and host communities using a continental-scale dataset from 15 African countries (figure 1*a*; electronic supplementary material, table S1).

It is important to note that most of our understanding of the ecology of AIV is derived from studies that have been conducted in boreal or temperate regions of the Northern Hemisphere (table 1). There is a knowledge gap in tropical regions, particularly in sub-Saharan Africa [3]. Yet, earlier studies have suggested that tropical regions may act as epicentres contributing to year-round AIV perpetuation in wild birds [10]. More recently, AIVs have been found circulating in wild birds across Africa [32–35] indicating that local environmental conditions are favourable for AIV transmission. However, the patterns of AIV prevalence observed in temperate or boreal regions cannot be directly transposed to the tropics where differences in host ecology, climate and seasonality may produce different dynamics of infection.

Eurasian (i.e. Palaearctic-breeding) migratory wildfowl winter in large numbers between September and March in Afro-tropical regions north of the equator [30,36] (figure 1*b*). All African regions also host an influx of Eurasian migrants from other waterbird groups including waders, gulls, terns, rails, herons and storks. In their wintering sites, Eurasian wildfowl mix with Afro-tropical wildfowl that reside year-round within sub-Saharan Africa (figure 1*c*). Afro-tropical wildfowl generally breed during or following the wet season but their breeding season is often much more extended than in Eurasian wildfowl, with laying periods stretching over 6–12 months for a given area [37]. In Afro-tropical regions, seasons are determined by rainfall rather than temperatures, which are higher and exhibit lower seasonal variation than in temperate regions. The duration of the wet season varies according to latitude with an asynchrony in the timing of rainfall between regions north and south of the equator (figure 1*d*). Most Afro-tropical wetlands, a key habitat and resource for wildfowl, experience extreme seasonal variations in their surface area: relatively short but intense rains and extensive river flooding can inundate vast floodplains [36], while high evaporation rates and human extraction of water drastically reduce the extent of wetlands during the dry season. The intensity of these seasonal drivers could be of great local importance: in the Inner Niger Delta in Mali, for instance, the surface area of seasonally flooded wetlands may be up to 20 times higher than the surface area of permanent wetlands [36].

Given the numerous differences between tropical and temperate ecosystems, we hypothesized that the mechanisms whereby host ecology and the environment influence AIV transmission in Afro-tropical regions should operate through ecological drivers derived from the context of Afro-tropical ecosystems. The presumably low environmental persistence of AIV under tropical climates might result in a predominant influence of ecological factors such as host density that are associated with inter-individual transmission (i.e. transmission through airborne droplets via the respiratory route or through short-lived viruses shed in the environment via the faecal–oral route), over climatic factors that are associated with environmental transmission (i.e. via long-term persisting virus in an environmental reservoir). In Palaearctic and Nearctic regions, the northern autumn peak in AIV prevalence consistently observed in ducks [5,10,17,18] has been related to the congregation of ducks at pre-migration and migration-staging sites, at a time when populations consist of a large proportion of first-year immunologically naive birds [5,10,25]. The resulting increase in the level of population immunity has been proposed to be a determinant of seasonal variations in AIV prevalence [5,19,25]. Analogously in the tropics, seasonality in AIV prevalence may be related to the congregation of wildfowl at permanent wetlands at the end of the dry season in response to the drying of wetlands. Another potential driver of AIV seasonality in Africa is the arrival of Eurasian migratory waterbirds, which represent a potential source of virus introduction (table 1).

Species variation in AIV prevalence is commonly reported between cohabiting wildfowl species [5,14]. A difference between Eurasian and Afro-tropical wildfowl in previous AIV exposure at breeding or migration-staging grounds may imply a difference in susceptibility to re-infection between these species when they co-habit in Africa. The higher AIV prevalence consistently reported in dabbling ducks (i.e. surface and shallow water foragers) of the *Anas* genus compared with other wildfowl species [3,5] suggests a potential heterogeneity in host competence among wildfowl species from different ecological guilds or taxonomic groups. These differences are commonly associated with foraging behaviour, with surface/shallow water feeders being more exposed to water-borne infection than divers or grazers ([3,5,19]; table 1). However, it may also result from a difference in receptivity to AIV infection among host species [19], determined by species-specific differences in the type of AIV receptors present on epithelial tissues [20]. Consequently, the proportion of the most competent host species present in a wildfowl community may have a substantial influence on the capacity of the community to perpetuate AIV.

While dabbling ducks in the Palaearctic and Nearctic are largely represented by *Anas* species, the dabbling ducks community in sub-Saharan Africa comprises a greater proportion of non-*Anas* species, particularly the abundant and widely distributed whistling ducks (*Dendrocygna* spp.). Afro-tropical regions north of the equator are characterized by a strong seasonal influx of Eurasian wildfowl, which are largely absent in the regions south of the equator. In contrast with East and Southern Africa, Afro-tropical *Anas* species are rare in West Africa, where *Anas* species are represented almost exclusively by Eurasian migratory ducks [30,36,37]; consequently, *Anas* species are scarce during half of the year in West Africa. These specificities and regional differences in the composition of the wildfowl community across sub-Saharan Africa provide the opportunity to tease apart in our continental study the respective influence of migratory patterns, foraging behaviour and taxonomy on species prevalence.

In this study, we used generalized linear mixed models and a model comparison approach to assess the ability of various ecological factors (table 2) to explain species, seasonal and geographical variations in AIV prevalence measured in wildfowl across Afro-tropical regions. We tested factors related to: (i) the probability of a wildfowl species being infected, including its migratory and foraging behaviours and taxonomic group; and (ii) the capacity of the local host community and environment to perpetuate the virus, including the host density (at species and wildfowl community level), the proportion of the potentially most competent species (Eurasian, dabbling or *Anas* species) in the wildfowl community, the climate (temperature and aridity indices) and the timing of sampling relative to the arrival of Eurasian migrants or to the dry season.

**Table 2.** Definition of the explanatory variables, presented in six categories of variables found to be associated and tested alternatively by permutation in models.

explanatory variables		definition (units)
species traits	geographical origin	Eurasian versus Afro-tropical spp.
	taxonomic group	*Anas* versus non-*Anas* spp.
	foraging behaviour	prim. dabbling versus non-prim. dabbling (i.e. mostly grazing or diving)
	origin × taxonomy	Eurasian *Anas*, Afro-tropical *Anas*, Afro-tropical non-*Anas* spp.
	origin × foraging	Eurasian prim. dabbling, Afro-tropical prim. dabbling, Afro-tropical non-prim. dabbling spp.
	taxonomy × foraging	*Anas* prim. dabbling, non-*Anas* prim. dabbling, non-*Anas-*non prim. dabbling spp.
wildfowl density	species	no. birds of the species sampled or of the entire wildfowl community per area of wetland (bird km^−2^)
	community	
wildfowl community composition	proportion of Eurasian spp.	percentage of birds from Eurasian, *Anas* or prim. dabbling spp. in the wildfowl community
	proportion of *Anas* spp.	
	proportion of dabbling spp.	
climatic conditions	maximum annual temperature	annual or monthly mean of maximum daily temperature for the month of sampling (°C)
	maximum month temperature	
	annual PET	annual or monthly mean of daily potential of evapo-transpiration (PET) (mm), computed as a function of radiations, humidity, air temperature and wind speed
	monthly PET	
	aridity index	ratio of annual rainfall to PET (i.e. deficit of available water)
season	timing relative to the arrival of Eurasian migrants	no. of days between the median sampling date and 1 September
	timing relative to the end of the dry season	no. of days between the median sampling date and the end of the previous dry season
sampling method		single cloacal, single oropharyngeal or both swabs

## 2. Material and methods

### (a) Sampling and avian influenza virus detection procedures

Free-living wildfowl were sampled between 2006 and 2009 at 16 sites, all permanent wetlands selected from among the most important waterbird areas within the study region (electronic supplementary material, table S1) [30]. Sampling was conducted on a different number of occasions between sites and years (electronic supplementary material, table S2), with at least two months between sampling occasions in any given site. All samples were collected using cotton swabs and immediately stored in cryovials containing a viral transport medium. Birds were tested for AIV infection using three distinct sampling methods that we distinguished in our subsequent analyses: a single cloacal swab, a single oropharyngeal swab or both cloacal and oropharyngeal swabs tested individually. Samples were analysed in different laboratories using a similar standard diagnostic procedure based on RNA extraction and real-time RT-PCR virus detection (see electronic supplementary material, SI methods, for a complete description of sampling and diagnostic procedures). We computed the observed prevalence for each species for each sampling occasion as the percentage of individuals found positive for AIV compared with the total number of birds tested.

### (b) Explanatory variables

Ground-based and satellite-based data were used to estimate the values of six categories of explanatory variables listed in table 2. Continuous variables (wildfowl density, community composition, climatic conditions and timing of sampling) were estimated for each sampling occasion. Details about data source are provided in electronic supplementary material (electronic supplementary material, SI methods).

### (c) Analysis

Measures of bird density were log-transformed and standardized, together with measures of proportion of wildfowl species and climatic variables, to have a mean of 0 and an s.d. of 1. We investigated the potential association between variables using the Pearson correlation coefficient for continuous variables and the phi coefficient for categorical variables. Following Graham [38], pairs of variables with a correlation coefficient greater than or equal to 0.28 were considered associated and were tested separately in models. Multi-collinearity was high among variables representing alternative measurements of wildfowl density, wildfowl community composition, climate or the timing of sampling but not between these categories of explanatory variables. The three variables related to species traits (origin, taxonomic group and foraging behaviour) also showed a strong association as some combinations of the categories of these variables were not represented in our sample. Non-*Anas* or non-primarily dabbling wildfowl species are rare among the Eurasian wildfowl wintering in sub-Saharan Africa [30,36,37] and were consistently absent from our samples. Similarly, there were no non-primarily dabbling *Anas* species in our samples. In order to nonetheless build models where the effects of two of these interdependent categorical variables reflecting species traits were simultaneously accounted for, we generated three composite variables that combined pairs of variables (table 2).

We investigated the relationships between AIV prevalence and explanatory variables using a generalized linear mixed model and assuming a binomial distribution. The 55 distinct sampling occasions were distributed over 16 sites and 4 years. There were thus several sampling occasions within a given year and usually within a given site. In order to tackle this potential pseudo-replication issue, a year and a site random effect were included in the models. The potential aggregations of infected birds within sampling occasions was also accounted for by incorporating the sampling occasion as a random effect nested within year and site. Finally, we included a random laboratory effect to account for a potential difference in diagnostic sensitivity among laboratories. Models were run with the ‘glmer’ function in the ‘lme4’ package in the R environment, using Laplace approximation of the maximum-likelihood and a logit link function. We used an information-theoretic procedure and the Akaike information criterion corrected for small sample sizes (AIC_c_) to compare models [39].

Our analysis consisted of three steps. We first selected among alternative explanatory variables that were found to be associated by testing them successively by permutation in models. We generated a first set of models that consisted of all the combinations of explanatory variables where a single variable was included for each of the six independent categories of variables (6 × 2 × 3 × 5 × 2 × 1 = 360 models). The relationships between prevalence and the two variables related to the timing of sampling (t) were considered as cyclic and were modelled using a cosine function of the form logit(prevalence) = *a* + cos(2*πt*/365). All the models considered at this step of the analysis included the fixed additive effects of six explanatory variables and the random effects of year, site, sampling occasions and laboratory. For each model *i*, we computed the Akaike weight (*ωi*) which can be interpreted as the likelihood that model *i* is the best model within the set in terms of trade-off between fit to the data and parsimony. For each independent category of explanatory variables, we selected for the next step of the analysis the variable that yielded the highest sum of Akaike weights (*Σωi*), computed for all models in the set in which that variable occurred [39].

In the objective of obtaining a minimal adequate model, i.e. a model including only important effects, and of assessing the statistical support associated with each random effect, we explored in a second step the random part of the model. We compared the variance components associated with each of the four random factors (year, site, sampling occasions and laboratory) in models that included as fixed effects the six explanatory variables previously selected. We iteratively removed from the model the random factors with the lower variance components by comparing AIC_c_s between models that included or excluded this random factor.

Finally, we evaluated the relative importance of each of the six independent explanatory variables retained after the initial selection by comparing *Σω*_*i*_ among models which included or excluded the effect of this variable. We created a second set of models which contained all possible combinations that could be generated by including or excluding each of the six independent explanatory variables as fixed effects (2^6^ = 64 models) and computed the *Σω*_*i*_ for each variable. We computed the coefficients of model parameters through model averaging across all models having *Δ*AIC_c_ < 2 (compared with the model with the lowest AIC_c_), weighting coefficients of model parameters by the model's Akaike weight and summing the weighted coefficients [39].

## 3. Results

### (a) Avian influenza virus detection

We sampled and tested a total of 8413 free-living wildfowl of 18 species (electronic supplementary material, table S3). AIV were detected in 3.3% (*n* = 278) of birds tested, in almost all countries (except Burkina Faso, Kenya and Mozambique), and in all species for which more than 31 birds were sampled (electronic supplementary material, table S3). The proportion of AIV-positive birds found was highly variable between species, sites and sampling occasions, reaching up to 14.7 per cent (*n* = 225) in garganey (*Anas querquedula*) in Mauritania in February 2006.

### (b) Ecological factors related to avian influenza virus prevalence

The initial selection among alternative explanatory variables within each independent categories of variables (table 2) indicated that the best predictors of AIV prevalence according to Akaike relative importance weights were (electronic supplementary material, table S4): (i) taxonomic group (*Σω*_*i*_ of the models with this variable = 0.50) compared with five other species traits tested separately or in pairs (all *Σω*_*i*_ ≤ 0.26); (ii) wildfowl density at the community level (*Σω*_*i*_ = 0.92) compared with the density at the species level; and (iii) timing of sampling relative to the arrival of Eurasian wildfowl (*Σω*_*i*_ = 0.84) rather than relative to the end of the dry season. The alternative variables related to wildfowl community composition or climate had Akaike weights of relatively similar importance: the proportion of Eurasian wildfowl (*Σω*_*i*_ = 0.40) and the mean maximum temperature of the month of sampling (*Σω*_*i*_ = 0.24) presenting the highest *Σω*_*i*_ were retained in the subsequent analyses.

The analysis of the dependence structure of our dataset indicated that the random effect of the site and the sampling occasion accounted for most of the variance of the random part, and the exclusion of the random effect of laboratory or year reduced the AIC_c_ of the mixed models (*Δ*AIC_c_ > 2). The site and the sampling occasion were thus included as the only random effects in all subsequent models.

The AIC_c_-based comparison procedure of the relative importance of each of the six independent explanatory variables retained after the initial selection indicated that four variables were important in explaining the variation in AIV prevalence (table 3). The high Akaike importance weights of the species taxonomic group (*Σω*_*i*_ =1), the density of the wildfowl community (*Σω*_*i*_ = 0. 88), the timing of sampling relative to the arrival of Eurasian migrants (*Σω*_*i*_ = 0.87) and the sampling method (*Σω*_*i*_ = 0.83) indicate that these variables occurred in all high ranking models. The three best-supported models (*Δ*AIC_c_ < 2) all included these four variables (table 3), and fitted the data adequately (Pearson *χ*^2^ goodness-of-fit test = 240.2–243.8, ddl = 265, *p* = 0.82–0.86, H0: ‘the model fits the data’ cannot be rejected). Inclusion of variables associated with the proportion of Eurasian wildfowl species (*Σω*_*i*_ = 0.32) or the mean maximum temperature of the month of sampling (*Σω*_*i*_ = 0.34) received much less support from the data.

**Table 3.** Summary of the three best-supported models (*Δ*AIC_c_ < 2) fitted to estimate variations in AIV prevalence in wildfowl in Afro-tropical regions, with coefficient estimates (±s.e.) and relative importance of selected explanatory variables (*Σ*ω**_*i*_). All models were fitted as generalized mixed effects models, with sampling site and occasion fitted as random intercept terms and other explanatory variables as independent fixed effect. *k*, number of estimable parameters; AIC_c_, Akaike's information criterion for small samples; *ω*, Akaike weights; *Σ*ω*i*, relative importance of each explanatory variable estimated by summing the Akaike weights of all models in the set where that variable occurred.

model	*k*	AIC_c_	*ω*	intercept	taxon. group non-*Anas* spp.	wildfowl community density	timing/arrival Eurasian migrants^a^	sampling method		maximum month temperature	proportion of Eurasian wildfowl spp.	random effects^b^	
								cloacal + oropharyngeal	single oroph.			site	occasion
1	8	265.6	0.31	−3.95 ± 0.35	−0.97 ± 0.17	0.53 ± 0.21	−0.68 ± 0.27	0.47 ± 0.23	−0.36 ± 0.47			0.67	0.43
2	9	267.0	0.15	−3.90 ± 0.35	−0.96 ± 0.17	0.52 ± 0.21	−0.71 ± 0.27	0.45 ± 0.23	−0.38 ± 0.47	−0.18 ± 0.22		0.70	0.41
3	9	267.5	0.12	−3.95 ± 0.35	−0.96 ± 0.17	0.51 ± 0.21	−0.67 ± 0.29	0.48 ± 0.23	−0.36 ± 0.47		0.12 ± 0.26	0.69	0.43
average				−3.94 ± 0.35	−0.97 ± 0.17	0.52 ± 0.21	−0.67 ± 0.27	0.47 ± 0.23	−0.36 ± 0.47	−0.18 ± 0.22	0.12 ± 0.26		
*Σω*i					1.00	0.88	0.87	0.83		0.34	0.32		

^a^Relationship modelled with a cosine function; ^b^random effect variance estimation.

**Figure 2.**
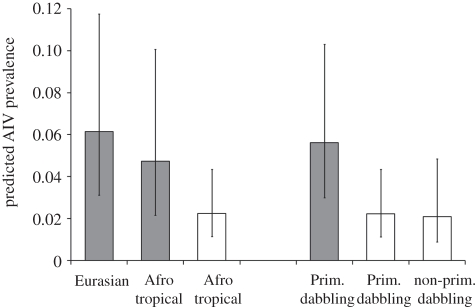
Mean AIV prevalence estimated for *Anas* (shaded bar) and non-*Anas* species (unshaded bar) of wildfowl belonging to distinct migratory groups and ecological guilds. Prevalences (95% CI, bars) were estimated for birds tested concurrently for cloacal and oropharyngeal samples, based on the highest rank model (table 3) after substituting the variable Taxonomic group by the composite variables Origin-Taxonomic group or Foraging behaviour-Taxonomic group. Other predictor variables were set to their mean value over the dataset.

The coefficients estimated from the top three models (table 3) indicate that prevalence was higher in *Anas* species than in non-*Anas* species. A similar difference was found between the two taxonomic groups when we accounted for the geographical origin or the main foraging behaviour of species (figure 2): *Anas* species showed a significantly higher prevalence than non-*Anas* species among Afro-tropical wildfowl (*Z* value = 2.45, *p* = 0.014) as well as among primarily dabbling wildfowl (*Z* value = 5.17, *p* < 0.001). These results are in agreement with the lower *Σω*_*i*_ found in the composite variables when compared with the variable taxonomic group tested separately (electronic supplementary material, table S4).

**Figure 3.**
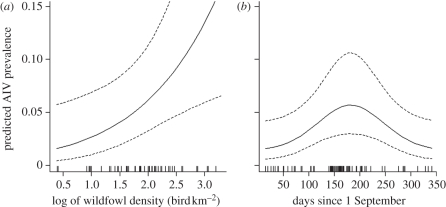
Predicted AIV prevalence (95% CI, dashed lines) for *Anas* species of wildfowl sampled across Afro-tropical regions in relation to the density of the wildfowl community and the timing relative to the arrival of Eurasian migrants (1 September used as a reference date). Prevalences were estimated for birds tested concurrently for cloacal and oropharyngeal samples, based on the highest rank model (table 3), with other predictor variables set to their mean value over the dataset. The distribution of data points is presented as rug plots along the *x*-axis (a vertical bar for each sampling occasion).

Prevalence was positively associated with the density of the wildfowl community (figure 3; rug plots illustrate the distribution of data points along the *x*-axis). Seasonal variation in AIV prevalence was related to the timing of arrival of Eurasian migrants: the prevalence progressively increased from the time when Eurasian migrants arrive in Afro-tropical regions (September) to peak at the end of their wintering period (February–March), and decreased after their departure (figure 3). Seasonal variation in AIV prevalence was poorly described by models including the effect of the timing relative to the end of the dry season. Substituting this variable with the timing relative to the arrival of Eurasian migrants substantially increased the AIC_c_ value of the best-supported model (*Δ*AIC_c_ = 4.8).

Finally, prevalence was higher for birds tested concurrently for both cloacal and oropharyngeal samples than in birds tested for a single cloacal sample (*Z* value = −2.20, *p* = 0.028). However, prevalence was similar between birds tested for a single cloacal or a single oropharyngeal sample (*Z* value = −0.66, *p* = 0.509). A similar trend was found for both the groups of *Anas* and non-*Anas* species (electronic supplementary material, figure S1). In addition, among birds tested for both cloacal and oropharyngeal samples individually (*n* = 3075), few birds were found positive concurrently for both types of sample (*n* = 6), while birds were found positive as frequently from cloacal samples only (*n* = 55) as from oropharyngeal samples only (*n* = 46) (McNemar test, *p* > 0.05).

## 4. Discussion

Our results indicate that variations in AIV prevalence in wildfowl at a continental scale were related to several host ecological factors operating at both species and community level, including the species taxonomic group, the local density of the wildfowl community and the season when Eurasian migratory birds winter in Africa. The timing relative to the dry season congregations, the composition of the local wildfowl community and the climatic variables were relatively poor predictors of AIV prevalence. It also appears that sampling the respiratory tract may be as important as sampling the digestive tract to detect AIV infection in wildfowl. We consider each of these points in more detail in the following paragraphs.

Prevalence was positively related to the density of wildfowl measured at the community rather than at the species level, suggesting aggregation of infection through interspecies mixing. The density of the wildfowl community varied widely between sites and seasons (up to 3 log units, electronic supplementary material, table S1), in relation to the seasonal variations in wetlands surface and the massive flux of Eurasian migratory wildfowl but also Afro-tropical wildfowl congregating at permanent wetlands during the dry season or, conversely, with the dispersal of birds to newly flooded wetlands after the onset of the wet season. The proportion of Eurasian wildfowl in the wildfowl community was poorly related to the variations in AIV prevalence suggesting that the influx of Eurasian wildfowl influences AIV transmission by increasing the local wildfowl density but that the geographical origin of birds may not matter much. We thus found no support to our initial prediction of a potential difference between Eurasian and Afro-tropical wildfowl in previous AIV exposure and susceptibility to re-infection, neither at the community level (proportion of Eurasian species) nor at the species level (species origin, figure 2).

Climatic conditions varied widely between season and between our study sites: these sites stretched over four aridity classes (from arid to humid), with local monthly and annual means of maximum daily temperatures varying between 20°C–39°C and 25°C–36°C, respectively (electronic supplementary material, table S1). Climatic variables associated with a reduced survival of the virus in the environment were, however, poorly related to AIV prevalence. Afro-tropical regions are characterized by mean monthly temperatures of greater than or equal to 20°C in all months (except in African highlands), in contrast with boreal and temperate regions that are characterized by mean monthly temperatures less than 20°C during at least eight months per year. Maximum daily temperatures in most Afro-tropical regions may be over a threshold throughout most of the year where high temperatures prevent the perpetuation of AIV in the environment by more than a few days [11]. The positive association, which we found between AIV prevalence and the local wildfowl density with no influence of climatic conditions, suggests a predominant role of direct inter-individual transmission via the respiratory route [8] or via short-lived viruses recently shed in the environment, rather than an indirect transmission via viruses persisting in the environmental reservoir. By contrast, in temperate regions theoretical models of AIV dynamics suggest a greater role for indirect environmental transmission than for density-dependent transmission [9,13].

Surprisingly, seasonal variations in prevalence were poorly related to the timing of congregation of wildfowl at the end of the dry season. In Palaearctic and Nearctic regions, concentration of wildfowl births into a short seasonal breeding period generates a pulse of immunologically naive birds into the host population [1,2]. The congregation of first-year susceptible birds during a relatively short autumn migration period, only a few months after the breeding season, probably increases the rate at which susceptible birds experience their first infection, producing a rapid increase in the level of population immunity. In Afro-tropical regions, extended breeding seasons produce a more gradual recruitment rate of juveniles into the host populations. The seasonal congregation of wildfowl in the dry season in the tropics is also more progressive than the northern migration flocking as it results from the progressive drying of wetlands while migration flocking results from a social gathering behaviour. These extended breeding seasons and progressive seasonal congregation may slow down the turnover rate of susceptible birds in the wildfowl community. The seasonality of AIV prevalence in our study (figure 3) was accordingly much less pronounced than in Europe (0–25%) [18] or North America (0–60%) [3]. This should reduce the controlling effect of population immunity on AIV transmission and promote a lower but continuous annual circulation as observed in a southern African wetland [34].

Our results indicated that AIV prevalence increases during the period when Eurasian migratory waterbirds (including non-wildfowl species) winter in sub-Saharan Africa and decrease after they migrate back to Eurasia. The arrival of Eurasian migrants constitutes a massive influx of hosts in the local waterbird community but also a potential source of AIV introduction. Eurasian wildfowl are largely absent in the regions south of the equator but large numbers of other Eurasian waterbird species, in particular shorebirds (Charadriiformes), winter in southern Africa. The role of shorebirds in the ecology of AIV is still unclear with highly contrasted results from Nearctic and Palaearctic regions [5,17]. A low prevalence has been reported globally in non-wildfowl species (less than 2%) [3,7] suggesting that they play a lesser role in the perpetuation of AIV, though locally shorebirds may have a significant role [7]. Phylogenetic analyses also indicate that inter-continental transfer of AIV genes, though occasional, do occur in shorebirds [40].

Difference in prevalence between species was better explained by the taxonomic group than by the foraging or the migratory behaviour of species. *Anas* species had higher prevalence than non-*Anas* species even when we account for difference in foraging behaviour or geographical origin of birds (figure 2). These results support the hypothesis [19] that there might be intrinsic differences between wild bird species, including between wildfowl taxonomic groups, in their receptivity to AIV infection. Dabbling ducks of the *Anas* genus are commonly reported to be more frequently infected than other wildfowl including grazing (*Anser*, *Branta* or *Cygnus* spp.) or diving wildfowl (*Aythya* spp.) [3,5,19]. Looking more closely at global AIV surveillance results reveal that non-*Anas* species of ducks that also forage primarily by dabbling in surface and shallow water (e.g. wood duck *Aix sponsa* and common shelduck *Tadorna tadorna*) have globally a lower prevalence than dabbling ducks of the *Anas* genus [3,5]. Similarly, differences in clinical disease and mortality to H5N1 HPAI virus infection has also been reported between *Anas* species (mallard *Anas platyrhynchos*, northern pintail *Anas acuta*, common teal *Anas crecca*) and non-*Anas* species of dabbling ducks (wood duck, muscovy duck *Cairina moschata*, ruddy shelduck *Tadorna ferruginea*, mandarin duck *Aix galericulata*) that had been concurrently experimentally inoculated [41–43]. A difference has been found between chickens and ducks in the distribution of sialic acid receptors of AIV on host epithelial tissues [20,44]; such a difference may also exist among different wildfowl species and limit interspecies transmission. Despite large differences in the proportion of *Anas* species in the wildfowl community between our study sites and seasons (1–96%, electronic supplementary material, table S1), variations in prevalence were poorly related to this variable. This suggests that the absolute rather than the relative number of birds from *Anas* species may influence AIV transmission.

In our study, the detection rate of AIV was similar in oropharyngeal and in cloacal samples and testing birds for both types of sample produced higher infection rates since birds were rarely found concurrently infected for both types. These results highlight the role of the respiratory tract for the replication of AIV. The traditional assertion that AIV replicate preferentially in the cells lining the intestinal tract of birds and are shed in their faeces [3,10] has been recently challenged by contrasted findings from several comparative studies reporting either a higher AIV detection rate from oropharyngeal samples [4,8,16] or from cloacal samples [26,45,46], or no difference in detection rates between these two sampling sources in wildfowl [4]. Moreover, experimental infection studies with H5N1 HPAI viruses have all evidenced a predominant oropharynx excretion in a diversity of wildfowl species (see the study of Gaidet *et al*. [27] for a review). The preferential site of replication may differ between species [4] and may be related to species' main foraging behaviour [8]. In our study, we found similar AIV detection rates in oropharyngeal and in cloacal samples in both *Anas* and non-*Anas* species (electronic supplementary material, figure S1). Though the exact role of a preferential oropharynx excretion on the dynamics of AIV transmission in wildfowl remains to be elucidated, the collection of oropharyngeal samples is essential for the field study of AIV in wild birds.

Our results provide a unique contribution to our understanding of the ecology of AIV in wild birds in tropical ecosystems but also offer a number of novel insights for understanding the general influence of seasonal fluctuations in animal density and migration on infectious disease dynamics. In addition, our approach illustrates the value of integrating ecology and epidemiology for understanding complex multi-host epidemiological systems. As our analysis shows, research at the interface between ecology and epidemiology could benefit hugely from cross-disciplinary inter-group data sharing and detailed empirical analyses of geographically diverse datasets.
